# DANCR Mediates the Rescuing Effects of Sesamin on Postmenopausal Osteoporosis Treatment via Orchestrating Osteogenesis and Osteoclastogenesis

**DOI:** 10.3390/nu13124455

**Published:** 2021-12-13

**Authors:** Zhengmeng Yang, Lu Feng, Haixing Wang, Yucong Li, Jessica Hiu Tung Lo, Xiaoting Zhang, Xuan Lu, Yaofeng Wang, Sien Lin, Micky D. Tortorella, Gang Li

**Affiliations:** 1 Stem Cells and Regenerative Medicine Laboratory, Department of Orthopaedics & Traumatology, Li Ka Shing Institute of Health Sciences, The Chinese University of Hospital, Hong Kong, China; 1155097544@link.cuhk.edu.hk (Z.Y.); wanghaixing1991@126.com (H.W.); yucongli@cuhk.edu.hk (Y.L.); Jessica020621@gmail.com (J.H.T.L.); zhangxt53@gmail.com (X.Z.); luxuan@link.cuhk.edu.hk (X.L.); sienlin@cuhk.edu.hk (S.L.); 2 Centre for Regenerative Medicine and Health, Hong Kong Institute of Science & Innovation, Chinese Academy of Sciences, Hong Kong, China; lufeng@link.cuhk.edu.hk (L.F.); yaofeng.wang@hkisi-cas.org.hk (Y.W.)

**Keywords:** sesamin, osteoporosis, Wnt/β-catenin, NF-κB, osteogenesis, osteoclastogenesis, DANCR

## Abstract

As one of the leading causes of bone fracture in postmenopausal women and in older men, osteoporosis worldwide is attracting more attention in recent decades. Osteoporosis is a common disease mainly resulting from an imbalance of bone formation and bone resorption. Pharmaceutically active compounds that both activate osteogenesis, while repressing osteoclastogenesis hold the potential of being therapeutic medications for osteoporosis treatment. In the present study, sesamin, a bioactive ingredient derived from the seed of Sesamum Indicum, was screened out from a bioactive compound library and shown to exhibit dual-regulating functions on these two processes. Sesamin was demonstrated to promote osteogenesis by upregulating Wnt/β-catenin, while repressing osteoclastogenesis via downregulating NF-κB signaling . Furthermore, DANCR was found to be the key regulator in sesamin-mediated bone formation and resorption . In an ovariectomy (OVX)-induced osteoporotic mouse model, sesamin could rescue OVX-induced bone loss and impairment. The increased serum level of DANCR caused by OVX was also downregulated upon sesamin treatment. In conclusion, our results demonstrate that sesamin plays a dual-functional role in both osteogenesis activation and osteoclastogenesis de-activation in a DANCR-dependent manner, suggesting that it may be a possible medication candidate for osteoporotic patients with elevated DNACR expression levels.

## 1. Introduction

Osteoporosis is a chronic bone disease with deteriorated bone tissue microstructure and reduced bone quality. Osteoporosis complications include fractures, chronic pain and secondary osteoarthritis, which may further increase the patients’ suffering and financial burden [1]. Currently, osteoporosis treatment involves the use of either suppressing the osteoclast resorption (e.g., bisphosphonates, calcitonin and estrogen) or promoting new bone formation agents (e.g., parathyroid hormone, fluoride) [2]. Besides these traditional approaches, natural small molecules were also proved to restrain osteoporosis. For example, phytohormones genistein, resveratrol and 8-prenylnaringenin were reported to be potential pharmaceutical products for osteoporosis treatment in animal models [3]. However, further investigations are needed to understand apply how these small molecules work and their underling regulation mechanism in osteoporosis treatment.

Promotion of osteogenic differentiation and repression of osteoclast formation were revealed to be effective treatment methods for osteoporosis therapy. As reported before, Wnt/β-catenin and NF-κB signaling pathways play major roles in these two differentiation processes, respectively [4]. In osteogenesis, upon Wnt signaling activation, the phosphorylation of β-catenin was inhibited, and its cytoplasm accumulation was increased. Increased β-catenin level could further promote Osx expression in mesenchymal progenitors and activate downstream Runx2 expression to promote early osteoblast differentiation [5]. While in osteoclastognenesis, canonical NF-κB signaling was induced by receptor activation of NF-κB ligand (RANKL) and tumor necrosis factor (TNF), which triggers IκB kinases complex activation and induces phosphorylation and degradation of IκBα as well as p65/p50 heterodimer release and nucleus translocation. The NF-κB downstream genes including c-Fos and nuclear factor of activated T-cells, cytoplasmic 1 (NFATc1) were both upregulated, and induce osteoclast precursor differentiation [6]. In general, both Wnt/β-catenin and NF-κB signaling pathways could be potential therapeutic targets for osteoporosis treatment.

Previous studies revealed that small molecules promoted osteogenesis by activating Wnt/β-catenin signaling, or repressed osteoclastognesis via deactivating NF-κB signaling activities. Li’s group reported that lithium released from lithium chloride/calcium phosphate cement could activate osteogenic differentiation and bone regeneration by activating Wnt/β-catenin signaling pathway, and further ameliorate osteoporosis progression [7]. Zhao et al. also identified a small molecule T63 which could activate osteogenic differentiation via BMP and Wnt/β-catenin signaling pathways and suppress osteoporosis [8]. On the other hand, several small molecules played their osteoprotective roles by repressing osteoclast formation coupled with the inhibition of NF-κB signaling activities. Berberine inhibited osteoclast formation and survival through suppression of NF-κB and Akt activation [9]. Quercetin also decreased RAW264.7 osteoclastogenesis via the inhibition of NF-κB and AP-1 activation [10]. Thummuri et al. found that abietic acid attenuated RANKL-induced osteoclastogenesis and LPS-triggered osteolysis by inhibiting the NF-κB and MAPK signaling activities [11]. Our group also found that another naturally occurring pentacyclic triterpenoid, Asiatic acid could inhibit osteoclastic differentiation and attenuate postmenopausal osteoporosis via targeting TGF-β and NF-κB signaling pathways [12]. In this study, our goal was to identify and characterize novel small molecules that could both activate Wnt/β-catenin and repress NF-κB signaling activities. If successful, this would allow us to develop novel therapeutic agents for osteoporosis that orchestrateboth osteogenesis and osteoclastogenesis simultaneously.

## 2. Result

### 2.1. Identification of Sesamin as an Up-Regulator of Wnt/β-Catenin and Down-Regulator of NF-κB Signaling

**Figure 1:**
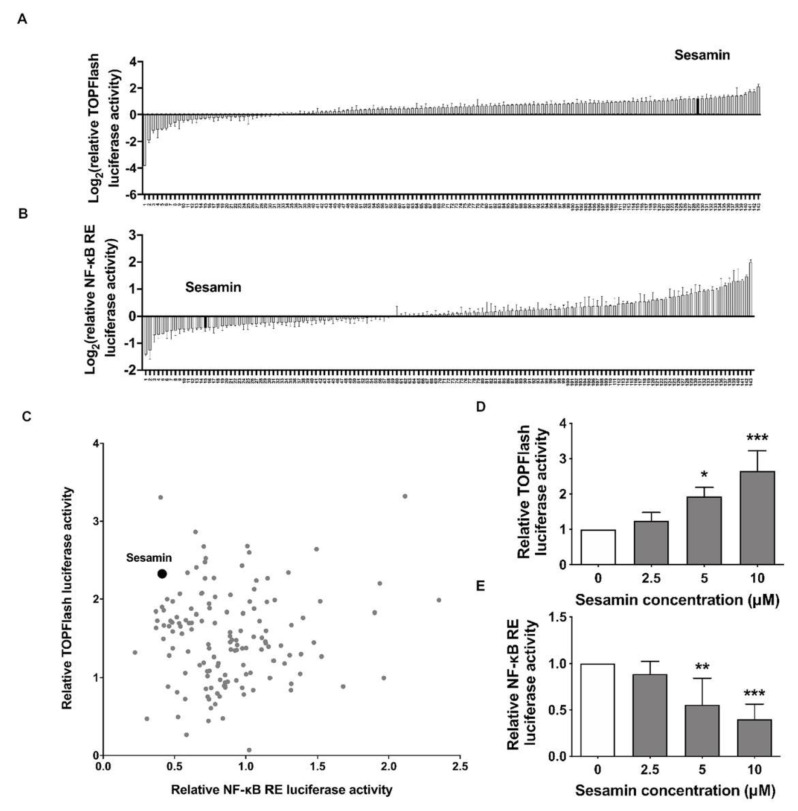
Screening of small molecules repressing luciferase activity of TOPFlash and NF-κB response element system. (**A**,**B**). Luciferase activities of TOPFlash (**A**) and NF-κB response element (**B**) reporter system upon small molecule library treatment. The fold changes of sesamin-regulated luciferase activity were highlighted. (**C**). Two-dimensional diagram with both luciferase reporter activities were plotted and sesamin was highlighted. (**D**,**E**). Luciferase activity of TOPFlash (**D**) and NF-κB response element (**E**) reporter system were decreased upon treatment with sesamin at different concentrations (*n* = 6; * *p* < 0.05, ** *p* < 0.01, *** *p* < 0.001).

A cell-based luciferase activity assay was applied to identify compounds that modulate osteogenesis and osteoclastogenesis activity simultaneously that could then be used in pharmacological proof of concept studies in vivo. To identify small molecules that could potentially stimulate osteoblast and repress osteoclast differentiation, concurrently, we designed a high-throughput screening system that allowed us to measure the luciferase activity of master regulators during osteogenesis and osteoclastogenesis. The TOPFlash vector reflects Wnt/β-catenin transcription activity, while NF-κB response element (RE) vector is responsive to NF-κB signaling activity. The luciferase activities of TOPFlash and NF-κB RE vectors upon treatment of small molecules were plotted, respectively (Figure 1A,B). The two-dimensional diagram was plotted to identify possible chemical compounds which showed a dual-regulating effect on both Wnt/β-catenin and NF-κB signaling activities (Figure 1C). Sesamin was observed in each diagram. After selecting sesamin as our lead hit, we confirmed that Wnt/β-catenin and NF-κB RE luciferase activity were increased and decreased, respectively upon sesamin treatment in a dose dependent manner (Figure 1D,E). These results indicated that sesamin had the capability of both up-regulating Wnt/β-catenin and down-regulating NF-κB signaling in vitro and made it a potential drug candidate for osteoporosis treatment in vivo.

### 2.2. Sesamin Promotes Osteogenic Differentiation of BMSCs by Activating Wnt/β-Catenin Signaling

**Figure 2:**
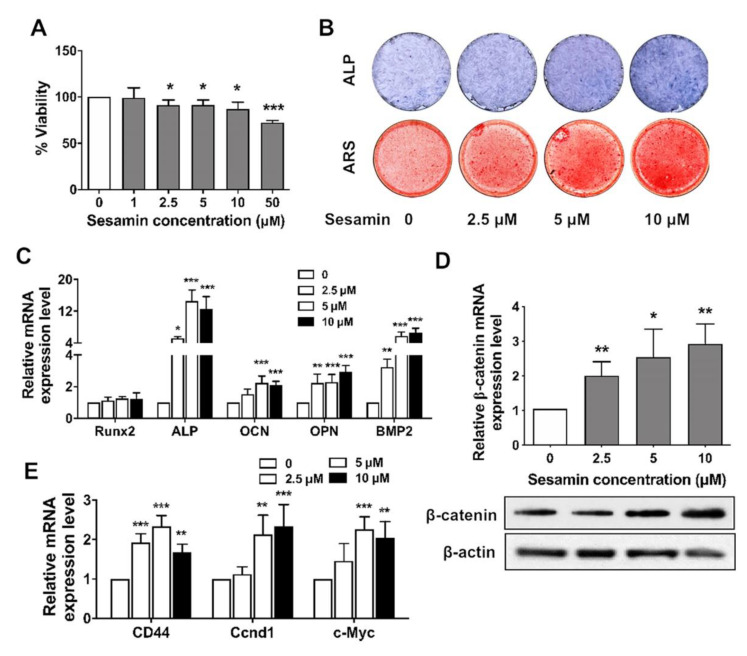
Sesamin promoted BMSCs osteogenesis via up-regulating Wnt/β-catenin signaling pathway. (**A**). Cytotoxicity assay of mouse BMSCs after treated with sesamin at different concentration for 3 days (*n* = 6; * *p* < 0.05, *** *p* < 0.001). (**B**)**.** The ALP staining at Day 3 and the ARS staining of calcified nodules at Day 7 after osteo-induction were performed. (**C**)**.** The mRNA expression level of osteogenic marker genes were detected by quantitative real-time PCR after treatment with sesamin after osteo-induction for 7 days (*n* = 6; * *p* < 0.05, ** *p* < 0.01, *** *p* < 0.001). (**D**)**.** Sesamin up-regulated the expression of β-catenin in mouse BMSCs at both mRNA and protein expression levels (*n* = 6; * *p* < 0.05, ** *p* < 0.01). (**E**)**.** The mRNA expression level of Wnt/β-catenin downstream genes in mouse BMSCs were significantly increased after sesamin treatment at different concentrations (*n* = 6; ** *p* < 0.01, *** *p* < 0.001).

We further tested the ability of sesamin to promote BMSCs osteogenesis by activating the Wnt/β-catenin signaling pathway. The viability of mouse bone marrow MSCs treated with sesamin revealed no significant effects at concentration ranging from 1 to 10 μM (Figure 2A). The BMSCs osteogenesis was induced with osteo-induction medium and treated with sesamin at different concentrations. The ALP staining at Day 3 and the ARS staining of calcified nodules were performed at Day 7 after osteo-induction. Increased ALP activity and stained mineralized nodules was revealed after treatment with higher concentrations of sesamin (Figure 2B). In addition, the mRNA expression level of osteogenesis marker genes including Runx2, ALP, OCN, OPN and BMP2 were significantly increased after sesamin treatment (Figure 2C). Sesamin up-regulated β-catenin expression in BMSCs at both mRNA and protein levels (Figure 2D). The mRNA expression levels of downstream genes mediated by Wnt/β-catenin signaling including CD 44, cyclin D1 and C-myc were all significantly increased after sesamin treatment (Figure 2E).

### 2.3. Sesamin Inhibits Osteoclastogenic Differentiation of RAW264.7 Cell by Deactivating NF-B Signaling Pathway

**Figure 3:**
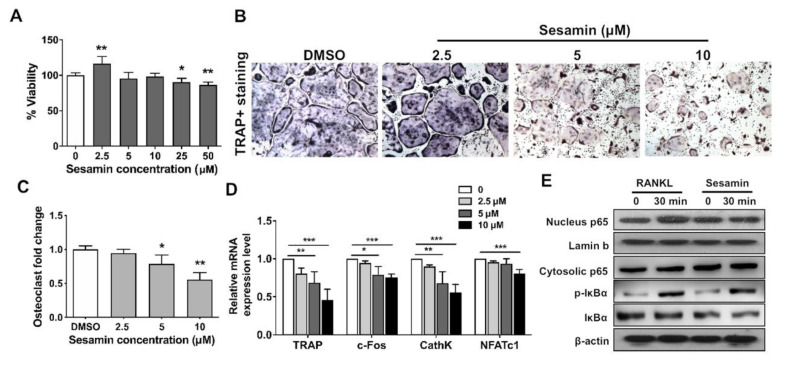
Sesamin inhibited osteoclastogenic differentiation of RAW264.7 cell by deactivating NF-κB signaling pathway. (**A**)**.** Cytotoxicity assay of RAW264.7 cells after treated with sesamin at different concentration for 3 days (*n* = 6; * *p* < 0.05, ** *p* < 0.01). (**B**)**.** TRAP staining assay at Day 5 after RANCL-induced osteoclastogenesis was performed. (**C**). Quantitative data of osteoclast fold change (*n* = 6; * *p* < 0.05, ** *p* < 0.01). (**D**)**.** The mRNA expression level of osteoclastogenic differentiation related markers measured by qRT-PCR (*n* = 6; * *p* < 0.05, ** *p* < 0.01, *** *p* < 0.001). (**E**). Analysis of NF-κB/p65 nucleus translocation and IκBα phosphorylation levels by Western blot analysis in RAW264.7 cells after cells were treated with sesamin for 24 h and treated with RANKL for 30 min.

The osteoclastogenesis of osteoclast progenitor cell line, RAW264.7 was induced with RANKL, with the co-treatment of sesamin. The cytotoxicity assay showed that sesamin had no effect on osteoclast precursor cells at concentrations from 0 to 10 μM (Figure 3A). Sesamin could effectively repress osteoclastogenesis of RAW264.7 in a dose-dependent manner as revealed by TRAP staining (Figure 3B,C). The mRNA expression level of osteoclastogenic differentiation markers including TRAP, c-Fos, CathK and NFATc1 were all decreased with increased sesamin concentrations (Figure 3D). Furthermore, IκBα phosphorylation and NF-κB p65 nucleus translocation in RAW264.7 decreased after sesamin treatment (Figure 3E). The result indicated that sesamin inhibited osteoclastogenesis by negatively regulating NF-κB signaling activity.

### 2.4. Osteogenic Role of Sesamin Is Dependent on DANCR Expression

**Figure 4:**
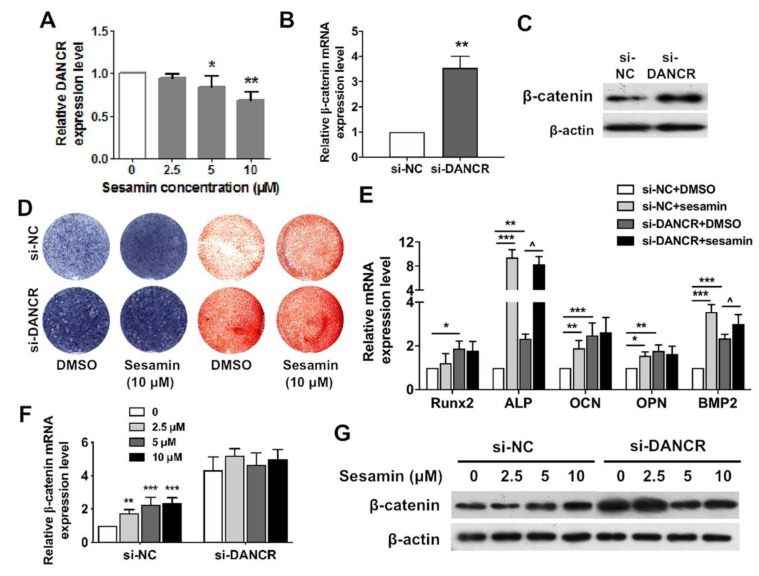
Knockdown of DANCR attenuated the Osteogenic effect of sesamin. (**A**). DANCR expression level decreased in BMSCs upon sesamin treatment (*n* = 6; * *p* < 0.05, ** *p* < 0.01). (**B**,**C**). DANCR knockdown upregulated the mRNA (**B**) and protein (**C**) expression level of β-catenin (*n* = 6; ** *p* < 0.01). (**D**,**E**). The BMSCs were DANCR knockdown and treated with sesamin during osteo-induction for 7 days. ALP activity, calcified nodules formation (**D**) and mRNA expression level of osteogenic marker genes (**E**) were examined by ALP activity assay, ARS staining and real-time PCR, respectively (*n* = 6; * *p* < 0.05, ** *p* < 0.01, *** *p* < 0.001, versus si-NC+DMSO group; ^ *p* < 0.05, versus si-DANCR+DMSO group). (**F**,**G**). Sesamin up-regulated the expression of β-catenin in mouse BMSCs at both mRNA (**F**) and protein (**G**) expression levels, this upregulation was attenuated upon si-DANCR treatment (*n* = 6; ** *p* < 0.01, *** *p* < 0.001).

Many lncRNAs have been demonstrated to serve as critical regulators in osteoporosis. In these studies, we propose that a ubiquitously expressed lncRNA in musculoskeletal tissues maybe a universal modulating factor and regulated by sesamin in osteoporosis. To identify the lncRNA targeted by sesamin , literature review was performed to screen out possible candidate lncRNAs that may regulate both osteoblast and osteoclast differentiation processes. We identified one lncRNA, DANCR which could exert both osteogenesis repressing and osteoclastogenesis promoting functions (Supplementary Table S1). Here, we demonstrated that DANCR expression levels was decreased in BMSCs upon sesamin treatment (Figure 4A). After si-DANCR treatment, β-catenin expression was decreased at both mRNA and protein levels in BMSCs (Figure 4B,C). In addition, BMSCs were also treated with sesamin at the concentration of 10 μM in the presence or absence of DANCR siRNA. Treatment of si-DANCR significantly increased the ALP activities, calcium nodule formation and osteogenesis marker expression of BMSCs during osteo-induction, while DANCR knockdown diminished the promoting effect of sesamin on osteogenesis compared with treatment of si-NC treatment groups (Figure 4D,E). These results demonstrated the link between DANCR and sesamin-mediated BMSCs osteogenesis. DANCR was also involved in promoting the effects of sesamin on mRNA and protein expression of β-catenin (Figure 4F), as well as the Wnt/β-catenin downstream genes that were upregulated by sesamin treatment (Figure 4G). Our results suggest that sesamin may exert its function via regulating DANCR expression, and DANCR might be the key regulator in sesamin mediated Wnt/β-catenin downregulation in BMSCs. However, further studies need be conducted to elucidate the precise regulation mechanism.

### 2.5. DANCR Mediates the Sesamin Repressed Osteoclastogneesis

**Figure 5:**
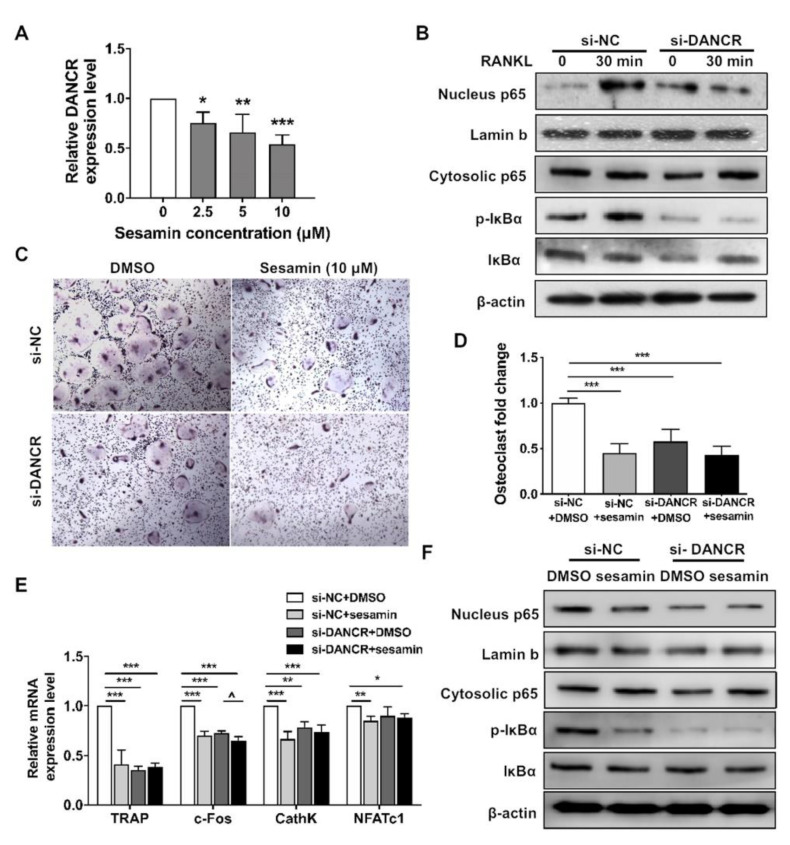
DANCR mediated sesamin-induced osteoclastogenesis of RAW264.7 cells. (**A**) DANCR was downregulated in RAW264.7 cells after treated with sesamin (*n* = 6; * *p* < 0.05, ** *p* < 0.01, *** *p* < 0.001). (**B**) Analysis of phosphorylation and non-phosphorylation levels of NF-κB/p65 and IκBα levels by Western blot analysis in RAW264.7 cells after DANCR siRNA knockdown and treated with RANKL for 30 min. (**C**)**.** RAW264.7 cells were DANCR knockdown and the TRAP staining assay was performed at Day 5 after RANKL-induced osteoclastogenesis. (**D**) Quantitative data of osteoclast fold change (*n* = 6; *** *p* < 0.001). (**E**) The mRNA expression level of osteoclastogenic differentiation related markers measured by real-time PCR (*n* = 6; * *p* < 0.05, ** *p* < 0.01, *** *p* < 0.001, versus si-NC + DMSO group; ^ *p* < 0.05, versus si-DANCR + DMSO group). (**F**) Analysis of NF-κB/p65 nucleus translocation and IκBα phosphorylation levels by Western blot analysis in RAW264.7 cells with/without DANCR knockdown after cells were treated with sesamin for 24 h and treated with RANKL for 30 min.

We also studied the mediating effect of DANCR on sesamin repressed osteoclastogenesis. DANCR expression in RAW264.7 cells was inhibited by sesamin treatment (Figure 5A). The IκBα phosphorylation and NF-κB-p65 nucleus translocation in si-DANCR transfected RAW264.7 cells were repressed upon RANKL treatment compared with si-NC control (Figure 5B). The TRAP staining result indicated that sesamin treated RAW264.7 cells formed more multinucleated TRAP-positive OC-like cells than control. While upon si-DANCR treatment, the frequency of TRAP-positive OC-like cell formation were both downregulated in sesamin-treated and control groups (Figure 5C,D). DANCR knockdown also abolished the repressive effect of sesamin on osteoclastogenesis marker mRNA expression (Figure 5E). Furthermore, DANCR siRNA treatment eliminated the downregulation effect of sesamin on IκBα phosphorylation and NF-κB-p65 nucleus translocation (Figure 5F).

### 2.6. Sesamin Attenuates Bone Loss in OVX Mice Osteoporosis Model via Down-Regulating DANCR Expression

**Figure 6:**
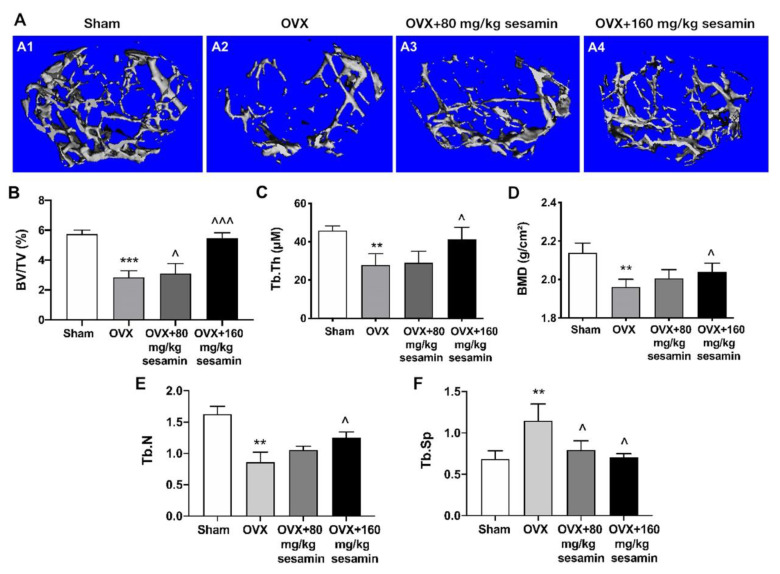
Microstructure properties of the trabecular bone of OVX mice after sesamin treatment. (**A**) Three-dimensional reconstruction of trabecular bone in the distal metaphysis of OVX mice after sesamin treatment. Scale bar, 100 μM. (**B**–**F**) Microstructure parameters including bone volume/tissue volume (BV/TV) (**B**), trabecular thickness (Tb.Th) (**C**), bone mineral density (BMD) (**D**), trabecular number (Tb.N) (**D**) and trabecular space (Tb.Sp) (**E**) of the trabecular bone. Data were shown as mean ± SD (*n* = 8; ** *p* < 0.01, *** *p* < 0.001, versus sham group; ^^^ *p* < 0.05, ^^^^^ *p* < 0.001. versus OVX group).

We next determined the efficacy of sesamin in osteoporosis in vivo. In the OVX-induced osteoporosis mouse model, two groups of mice were administrated with two different doses of sesamin daily (80 mg/kg and 160 mg/kg, designated as sesamin-L and sesamin-H respectively) for two months. The femurs of the sacrificed mice were collected for analysis. Micro-CT analysis revealed that the altered cancellous bone microstructure at the distal site of the femur after OVX were significantly improved by sesamin treatment (Figure 6A). The bone volume/total volume (BV/TV), trabecular thickness (Tb.Th.), trabecular number (Tb.N) and bone mineral density (BMD) in femurs were significantly lowered, and trabecular space (Tb.Sp.) was dramatically increased in the OVX model, while both low- and high-dosage sesamin treatment could reverse these trends compared with the control group (Figure 6B–F). The results indicate that sesamin significantly rescues bone mass loss incurred by OVX.

**Figure 7:**
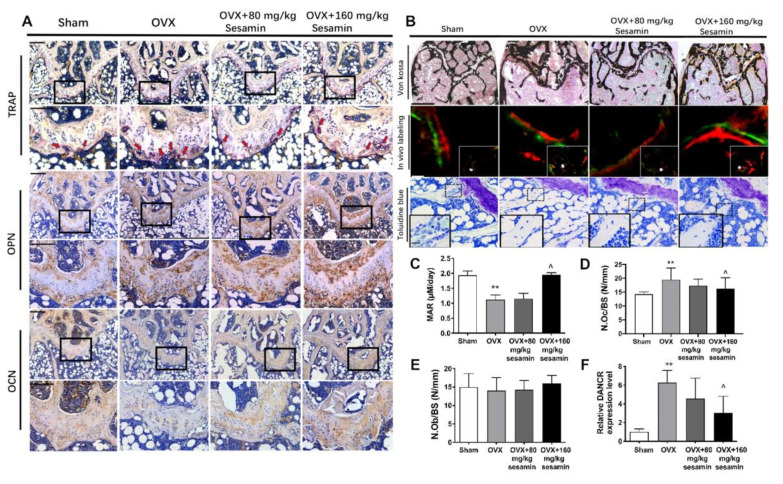
Histomorphometry of the distal femur from OVX mice upon sesamin treatment. (**A**) Representative images of femoral metaphysis with IHC staining using TRAP, OCN and OPN antibodies. Red arrow indicates the TRAP positive cells. Scale bar: 200 μM. (**B**) Von Kossa staining, in vivo double labels and Toluidine blue staining of the distal femur sections. Scale bar: 400 μM for Von Kossa, 100 μM for in vivo labeling and Toluidine blue staining. (**C**–**E**) Histomorphometric analysis of distal femur sections including MAR (mineral apposition rate) (**C**), N.Oc/BS (number of osteoclast per bone surface) (**D**) and N.Ob/BS (number of osteoblasts per bone surface) (**E**). (**F**) Serum level of DANCR was revealed in OVX mice upon sesamin treatment. Data were shown as mean ± SD (*n* = 8; ** *p* < 0.01, versus sham group; ^^^ *p* < 0.05. versus OVX group).

We further performed histological and histomorphometrical analysis of the distal femur metaphysis. TRAP staining indicated that the osteoclast amount in the trabecular bone surfaces were reduced upon OVX surgery. Sesamin treatment increased the TRAP-positive staining area (red arrows) in the distal head of the femurs compared with the OVX group, at both low and high dosage. Immunostaining showed that OCN and OPN expression were reduced in the OVX group as compared to the Sham group, while their expression was partially enhanced in the Sesamin-L and Sesamin-H groups (Figure 7A). The von Kossa and toluidine blue staining also showed the impaired bone mass in the OVX group was partially rescued by sesamin as observed in both Sesamin-L and Sesamin-H groups. The calcein double-labeling analysis displayed an accelerated bone growth rate upon sesamin treatment (Figure 7B). Bone parameter quantitative analysis indicated that the rate of osteogenesis (mineral appositional rate, MAR) were decreased (Figure 7C), while the number of osteoclast/bone surface (N.Oc/BS) was increased in OVX group as compared with the Sham group (Figure 7D), and the levels of these parameters were partially reversed by the treatment of sesamin at both low and high dosage after 8 weeks. However, osteoblast/bone surface area (N.Ob/BS) showed no significant difference among different groups (Figure 7E). Furthermore, serum DANCR levels were increased upon ovariectomy, and sesamin treatment could downregulate serum DANCR levels to some extent (Figure 7F).

## 3. Discussion

In the present study, sesamin was found to promote osteogenesis by activating Wnt/β-catenin while repressing osteoclastogenesis via de-activating NF-κB signaling pathways. Further investigation of the underlying mechanisms demonstrated that DANCR was involved in sesamin mediated osteogenesis and osteoclastogenesis , respectively. Knockdown of DANCR could abolish the stimulating effect of sesamin on Wnt/β-catenin as well as its repressive effect on NF-κB signaling activities. Therefore, our study provides a new mechanism of sesamin in osteoporosis prevention and may represent a therapeutic candidate for osteoporotic patients.

For osteoporosis treatment, several pharmacological medications could be used with the aim of increasing bone mass and strength by inhibiting bone resorption or promoting bone formation [13]. However, the widely used bone-resorbing bisphosphonate inhibitors decrease bone degradation while reducing bone formation simultaneously [14]. The parathyroid hormone (PTH) analogues, which were also used/tried for osteoporosis treatment, not only accelerated bone synthesis, but also promoted bone degradation in osteoporotic patients [15]. The recently approved anti-osteoporotic drugs, odanacatib and romosozumab also display uncoupled effects on bone formation and resorption. They were both classified as bone resorption inhibitors via inhibiting cathepsin K activity and neutralizing sclerostin (SOST), respectively. While odanacatib only produced a transient increase in bone formation at the early stage of osteoporosis, romosozumab exhibited only a moderate preservation effect of bone formation [16,17]. It is very attractive to explore a new class of dual functional therapeutic agents for osteoporosis that can inhibit both bone resorption and while promoting bone formation, synchronously. In our study, we identified sesamin from 143 potential pharmacologically active compounds as possible dual-functional candidates for osteoporosis treatment. Sesamin and sesamolin were the major beneficial nutrients extracted from the seed of Sesamum Indicum [18]. Phitak et al. reported that sesamin could prevent osteoarthritis by specifically controlling the Δ5-desaturase in polyunsaturated fatty acid biosynthesis [19]. Orawan et al. also revealed the effect of sesamin on stimulating osteoblast differentiation by activating p38 and ERK1/2 MAPK signaling pathways [20]. Furthermore, sesamin promotes BMSCs osteogenesis via activating Wnt/β-catenin signaling pathway and protects rat from osteoporosis [21]. Besides, sesamin displayed inhibitory effects on osteoclast differentiation and function. It also decreased the resorption pits and the collagen released from the bone slices [22]. However, a systematic investigation of the underlining mechanism by which sesamin simultaneously regulates osteoblast and osteoclast formation in the osteoporotic process has not been properly investigated. In our study, we characterize sesamin as a small molecule simultaneously activate Wnt/β-catenin signaling and osteogenesis while repressing NF-κB and osteoclastogenesis, which makes it an ideal compound for osteoporosis treatment. In the previous study, Liu et al. found that wedelolactone could promote osteoblastogenesis by regulating Wnt/β-catenin signaling while repressing osteoclastogenesis by targeting the NF-κB/c-fos/NFATc1 pathway. Administration of wedelolactone ameliorated OVX-induced bone loss [23]. This study helped define our study strategies to characterize the dual-functionality of chemicals for osteoporosis treatment by synchronously regulating osteogenesis and osteoclastogenesis processes.

In recent years, the noncoding RNAs (ncRNAs) were revealed to be largely involved in the regulation of osteoporosis. Among them, long non-coding RNAs (lncRNAs) with a length of over 200 bases have recently been identified as novel regulators of osteoporotis related gene activity [24]. The regulatory roles of lncRNAs in bone homeostasis and osteoporosis have been recognized. For example, H19, linc-ROR, HOTAIR and MODR were reported to be involved in bone formation [25,26,27]. The lnc-ob1 was reported to activate Osx expression in osteoblast and ameliorate bone loss in OVX mice [28]. However, the biological role of lncRNA in regulating osteoclast differentiation in vitro and controlling bone resorption in vivo is less studied yet. Wang’s group identified the functional motif a lncRNA Nron that could regulate ERα stability to prevent bone loss [29]. In the present study, we have identified the lncRNA DANCR a key factor for sesamin-mediated osteogenesis and osteoclastogenesis, as well as the regulator in osteoporosis pathogenesis. DANCR is a recently identified 8.3-kb lncRNA located on Chromosome 4, consisting of five exons. DANCR was revealed to repress osteogenic differentiation in osteoporosis through inhibiting β-catenin expression [30]. In addition, DANCR expression was found upregulated in circulating monocytes, and increase their bone-resorbing activities in osteoporotic patients and identified as a potential biomarker of postmenopausal osteoporosis [31]. In our study, we found that DANCR was a key factor regulated by sesamin . Sesamin could activate Wnt/β-catenin and deactivate NF-κB signaling pathway by downregulating DANCR expression, and further promoting osteogenesis and repressing osteoclastogenesis, respectively. Ma et al. also reported that DANCR upregulates AXL expression and activates PI3K/Akt/NF-κB signaling pathway in glioma cells, which further strengthened our finding that sesamin represses NF-κB signaling activity via the downregulation DANCR expression [32]. Our study demonstrates the unique participation of lncRNA DNACR in sesamin rescued bone homeostasis in osteoporosis.

Osteoporosis is a common disease mainly resulting from the imbalance of bone formation and bone resorption. In our study, we found that upon ovariectomy, the bone resorption in the trabecular bone of the mouse femur was enhanced while the bone formation was inhibited as revealed by a significant reduction of bone microstructure and reduced bone mechanical properties. The expression level of TRAP, a specific marker of bone resorption was increased, while OPN and OCN, the late-stage markers of osteoblastic differentiation and bone regeneration were deceased in OVX mice compared with Sham control. Sesamin treatment could effectively reverse this trend of OVX-induced bone loss. Previous studies also revealed a significant upregulation of DANCR in blood mononuclear cells (MNCs), and DANCR could promote the expression of IL-6 and TNFα expression and elevated the bone resorption activity of MNCs in postmenopausal women [31]. In our study, the serum level of DANCR was also upregulated upon OVX application, while sesamin treatment could obviously block it as well. Sesamin was proposed to be an effective treatment option for postmenopausal osteoporosis patients with aberrantly DANCR expression levels. However, there are several limitations in our studies. Although we have elucidated the involvement of DANCR in the regulation mechanism of sesamin on Wnt/β-catenin and NF-κB signaling in in vitro whether these two signaling pathways are predominant in the observed efficacy of sesamin in osteoporosis is yet to be explored in in vivo OVX mouse models. In addition, the long-term effects of seamin in osteoporosis in human should also be evaluated due to the disease progress difference of OP between mouse and human.

In summary, our results show that the efficacy of sesamin in osteoporosis is through the regulation of DANCR expression and the downstream Wnt/β-catenin and NF-κB signaling pathways during osteogenesis and osteoclastogenesis, respectively. This study elucidates a new mechanism/role of DANCR in mediating the beneficial effects of sesamin in postmenopausal osteoporosis treatment and sheds light on developing sesamin as a novel agent for osteoporotic patients, especially for postmenopausal women with high serum DANCR levels. However, although sesamin is considered as a nutraceutical in foods and supplement products, intake of sesamin from daily foods could be not sufficient for anti-osteoporosis activity because of its poor solubility and chemical instability, which results in low bioavailability. Thereafter, the dosage of sesamin we applied for osteoporosis treatment is much higher than the clinical dosage of odanacatib and romosozumab. The physiochemical properties of this natural molecule will be studied as to optimize its delivery capability and to expand its application in clinical practice.

## 4. Materials and Methods

### 4.1. Small Molecule Library

Selleck natural product library was purchased from Selleck Chemicals (Harris County, TX, USA). This collection contains 143 active compounds of nature product selected by a team of medicinal chemists and pharmacists for their high structural and pharmacological diversity, high cell permeability and effective bioavailability and reliable safety. The compounds were supplied as DMSO solution at the concentration of 10 mM.

### 4.2. Cell Culture

The human embryonic kidney 293 (HEK293) cells and mouse mononuclear macrophage-like cell line RAW 264.7 were obtained from ATCC and was cultured with Dulbecco’s Modified Eagle Medium (DMEM) supplemented with 10% fetal bovine serum (FBS) and 1% penicillin/streptomycin/neomycin (PSN). The mouse bone marrow mesenchymal stem cells (BMSCs) were isolated and kept in our laboratory as previously described [33]. The BMSCs were cultured in Minimum Essential Medium Alpha (MEMα) plus 10% FBS and 1% PSN. BMSCs were characterized using flow cytometry for phenotypic markers including MSC positive markers CD44-FITC, CD90-PE and negative markers CD31-FITC and CD45-FITC following the previous mentioned. All cells were kept in a humidified 5% CO_2_ incubator at 37 °C.

### 4.3. Transient Transfection

The DANCR small interfere RNAs (si-DANCR) and control siRNAs (si-NC) were synthesized by GenePharma (Shanghai, China). Wnt/β-catenin response element (RE) luciferase reporter vector TOPFlash, NF-κB response element (RE) luciferase reporter vector and Renilla luciferase control reporter vectors were purchased from Upstate Cell Signaling (Cell Signaling Technology, Albany, NY, USA). The siRNA as well as plasmids were transfected using Lipofectamine 3000 (Invitrogen, Carlsbad, CA, USA) following the manufacturer’s instructions.

### 4.4. Luciferase Activity Screening

A high-throughput luciferase activity screening method was applied to screen out the putative natural products which regulate promoter luciferase activities. The HEK293 cells were seeded in 24-well plates at the density of 5000 cells/well. When the cells were 80% confluent, they were co-transfected with Renilla vector together with TOPFlash or NF-κB RE reporter vector, respectively. The cells were then treated with chemicals from Selleck small product chemical library at the final concentration of 10 μM. After one-day treatment, the cells were harvested and applied to dual-luciferase assay according to the instructions of dual-luciferase assay reagent (Promega, Madison, WA, USA) with some modifications. In brief, cell lysate was placed into a PerkinElmer VictorTM X2 2030 multilateral reader (PerkinElmer, Waltham, MA, USA) to measure the firefly luciferase activity as well as the renilla luciferase activity. The ratio of firefly luciferase to renilla activity in each sample was revealed as a measurement of the normalized luciferase activity. Triplicated experiments were performed.

### 4.5. Cell Viability Assay

The effect of sesamin on the viability of BMSCs and RAW246.7 cells were measured by using 3-(4,5-Dimethylthiazol-2-yl)-2,5-diphenyltetrazolium bromide (MTT) assay. In brief, BMSCs (5 × 10^3^/well) or RAW246.7 cells (5 × 10^4^/well) were seeded in 96-well plate and cultured overnight. The cells were then treated with various concentrations of sesamin for 72 h. After that, 10 μL of 0.5 mg/mL MTT was applied into each well and incubate at 37 °C for additional 4 h. After incubation, the supernatant was discarded and 100 μL DMSO was applied for solubilization of formazan crystals. The light absorbance at 570 nm was measured with a Spectramax Gemini dual-scanning microplate reader (Molecular Devices, San Jose, CA, USA).

### 4.6. Osteogenic Differentiation

To induce the BMSCs osteogenesis, the mouse BMSCs were seeded in 12-well plates. When reached 90% confluency, the osteogenesis induction was applied to the cells by using StemPro^TM^ Osteogenesis Differentiation Kit (Life Technologies, Carlsbad, CA, USA) with the addition of sesamin (2.5, 5, 10 μM). The ALP activity assay was performed 7 days post-osteogenesis induction following the previous protocol [26]. The cells were then stained with ALP substrate solution [0.5 mg nitroblue tetrazolium chloride (NBT) and 0.25 mg 5-bromo-4-chloro-3′-indolyl phosphate p-toluidine salt (BCIP) in 1 mL ALP buffer] for 1 h at 37 °C in dark place. The Alizarin Red S (ARS) staining was performed 14 days since the beginning of osteo-induction. The cells were stained with 2% ARS staining solution for 10 min. The stained plates were scanned by Epson launches Perfection V850 (Seiko Epson, Suwa-shi, Nagano, Japan).

### 4.7. Osteoclastogenesis

Raw 264.7 cells were seeded in 24-well plate at the density of 5 × 10^4^ cells/cm^2^. RANKL (50 ng/mL) and sesamin (2.5, 5, 10 μM) or DMSO were applied to the cells for the osteoclastogenesis induction. After 5-day induction, the cells were fixed in 4% paraformaldehyde for 10 min and stained with Tartrate-resistant acid phosphatase (TRAP) activity kit according to the manufacturer’s instructions (386A, Sigma-Aldrich, St Louis, MO, USA). The TRAP-positive multinucleated (nuclei > 3) cells were scored as osteoclasts under a light microscope.

### 4.8. Mouse Ovariectomy (OVX)-Induced Osteoporosis Model

All animal experiments were approved by the Ethics Committee of Chinese University of Hong Kong and performed in accordance with the Code of Ethics of the World Medical Association. Thirty-two C57BL/6 mice (female, 14 weeks old, 20–25 g) were subjected to either ovariectomy (OVX, *n* = 24) or sham (Sham, *n* = 8) surgery. OVX surgery was performed by removing the bilateral ovaries as described previously [12]. The same procedure was carried out except for the removal of the ovaries in the sham groups. After 7 days recover, the OVX mice were randomly divided into 3 groups (8 mice/group) and orally administered with saline (100 μL/day), low dosage sesamin (100 μL, 80 mg/kg/day) or high dosage sesamin (100 μL, 160 mg/kg/day) daily for 2 months. All mice were subcutaneously injected with Calcein green (10 mg/kg) and xylenol orange (90 mg/kg) at days 14 or 4, respectively before sacrifice. The mice were then killed under anesthesia, and the femurs and serum from mice of each group were collected for further analysis.

### 4.9. Micro-Computed Tomography (μCT) Analysis

To analyze the microstructure of distal femoral metaphysis, the high-resolution micro-computed tomography (μCT) 40 (Scanco Medical, Switzerland) with a voltage of 70 kV and 10.5 μm isotropic resolution was used. The gray-scale images were employed to perform three-dimensional (3D) reconstruction of the mineralized tissues using a fixed threshold (158 mg hydroxyapatite/cm^3^) as described before [33]. Parameters including BV/TV, BMD, Tb.Th., Tb.Sp. and Tb.N. were recorded for analysis.

### 4.10. Histology and Immunohistochemistry

The femurs were 10% buffered formalin fixed, decalcified by 10% EDTA at 37 °C for 2 weeks and then embedded in paraffin. The paraffin-embedded tissues were sectioned at 7-μm thickness and performed with TRAP staining (Sigma-Aldrich, St Louis, MO, USA), Toluidine blue O staining (Sigma-Aldrich, St Louis, MO, USA) and IHC staining according to manufacturer’s instruction. For IHC staining, the sections were incubated with primary antibodies including rabbit anti-OCN (1:100, ab93876, Abcam, Cambridge, MA, USA) and rabbit anti-OPN (1:200, ab8448, Abcam, Cambridge, MA, USA) overnight at 4 °C. The horseradish peroxidase-streptavidin system (Dako, Carpinteria, CA, USA) was used for signal detection which followed by counterstaining with hematoxylin.

To process the un-decalcified femur specimens, the femurs were fixed by 10% buffered formalin overnight and embedded in methyl methacrylate. The samples were sectioned at 7-μm thickness by RM2155 hard tissue microtome (Leica, Wetzlar, Germany). The sections were stained by the von Kossa/nuclear fast red method as well as Masson-Goldner trichrome staining technique following the previous protocol [34]. The unstained 7-μm sections were used for dynamic histomorphometric analysis by a semi-automatic digitizing image analysis system (OsteoMetrics, Atlanta, GA, USA) as mentioned previously [35].

### 4.11. RNA Extraction and Real-Time PCR

Total RNA from tissue culture was extracted with TRIzol™ reagent (Life Technologies, Gaithersburg, MD, USA). RNA from mice serum was extracted using TRIzol LS Reagen (Life Technologies, Gaithersburg, MD, USA). The cDNA was reversely transcribed by using PrimeScript RT Master Mix (TaKaRa, Otsu, Japan) according to the manufacturer’s protocols. Real-time PCR was performed using Power SYBR Green PCR Mix (Life Technologies, Gaithersburg, MD, USA) on the QuantStudio 7 Flex Real-time PCR system (Applied Biosystems, Foster City, CA, USA). The target gene expression level was calculated by the 2^−ΔΔCq^ method and normalized by using GAPDH expression level. The primers used were listed in Supplementary Table S2.

### 4.12. Western Blot Analysis

Total protein of harvested cells was lysed by RIPA buffer (Sigma-Aldrich, St Louis, MO, USA) which supplemented with protease inhibitor cocktail (Roche, Basel, Switzerland). Collection of cytoplasmic and nuclear protein was performed using NEPER Nuclear and Cytoplasmic Extraction Reagents (Pierce Biotechnology, Radford, IL, USA) according to the manufacturer’s protocol. Whole cell extracts, cytoplasmic fractions or nuclear extract protein samples were electrophoresed on the SDS-PAGE gel and electroblotted onto a PVDF membrane as described in previous studies [36]. The PVDF membrane was then blocked with 5% non-fat milk and probed with the listed antibodies: β-catenin (1:3000, 610153, BD Biosciences, USA), phospho-IκBα (1:1000, CST-2859, Cell Signaling Technology, Albany, NY, USA), IκBα (1:3000, CST-4814, Cell Signaling Technology, Albany, NY, USA), p65 (1:3000, CST-3034, Cell Signaling Technology, Albany, NY, USA ), β-actin (1:3000, Sc-47778, Santa Cruz Biotechnology, Dallas, TX, USA), Lamin B (1:3,000, D4Q4A Cell Signaling Technology, Albany, NY, USA). The results were visualized on the X-ray file by Kodak film developer (Fujifilm, Tokyo, Japan).

### 4.13. Statistical Analysis

All data were presented as mean ± standard deviation. Experiments were repeated independently at least three times, and representative data are shown. The statistical significance between two groups was calculated by unpaired, two-tailed Student’s *t*-test for parametric data and by Mann–Whitney U test for non-parametric data analysis. The analysis was performed with GraphPad Prism 8 (GraphPad Software, San Diego, CA, USA). *p* < 0.05 was revealed as significant difference.

## Supplementary Materials

The following are available online at https://www.mdpi.com/article/10.3390/nu13124455/s1, Table S1: Summary of lncRNAs involved in osteogenesis and osteoclastogenesis processes, Table S2: Primers used for vector construction and Real-time PCR assays.
