# Supplementary material for: DANCR Mediates the Rescuing Effects of Sesamin on Postmenopausal Osteoporosis Treatment via Orchestrating Osteogenesis and Osteoclastogenesis
Source: Nutrients. 2021 Dec 13;13(12):4455. doi: 10.3390/nu13124455 (PMC8704418; doi:10.3390/nu13124455)
Supplement: Supplementary file 1 [file nutrients-13-04455-s001.zip › nutrients-1499461-supplementary.pdf]

Supplementary Table S1. Summary of lncRNAs involved in osteogenesis and osteoclastogenesis processes

**Anti-osteogenesis**	**Pro-osteogenesis**
NR2F2-AS1, XIST, MIAT, MIR31HG, HOXA-AS3, HOTAIR, ORLNC1, **DANCR**	PGC1-OT1 AK141205, Linc-ROR, H19, NTF3, MEG3, lnc-OG, MALAT1, MDOR, BMNCR, lnc-OB1
**Anti-osteoclastogenesis**	**Pro-osteoclastogenesis**
NRON, NONMMUT037835.2	AK077216, **DANCR**, Gm12310, Gm12308, PBOV-1, lncRNA-Jak3, AK131850, LINC00311

Supplementary Table S2. Primers used for vector construction and Real-time PCR assays

**Primers for Real-time PCR**				
Gene ID	Accession No.	F/R	Sequence	Product size (bp)
mRunx2	NM_001146038	F<br>R	ACACCGTGTCAGCAAAGC<br>GCTCACGTCGCTCATCTTG	99
mALP	NM_007431	F<br>R	ACTGCGCTCCTTAGGGCT<br>GGCAGCGTCAGATGTTAATTG	104
mOCN	NM_007541	F<br>R	GGTAGTGAACAGACTCCGGC<br>CAAGCAGGGTTAAGCTCACA	96
mOPN	NM_001204201	F<br>R	ATTTGCTTTTGCCTGTTTGG<br>TGGCTATAGGATCTGGGTGC	109
mBMP2	NM_007553	F<br>R	AACACCGTGCGCAGCTTCCATC<br>CGGAAGATCTGGAGTTCTGCAG	143
mβ-catenin	NM_007614	F<br>R	GAGCCGTCAGTGCAGGAG<br>CAGCTTGAGTAGCCATTGTCC	129
mCD44	NM_009851	F<br>R	CAAGTTTTGGTGGCACACAG<br>AGCGGCAGGTTACATTCAAA	95
mCCND1	NM_001379248	F<br>R	TCCTCTCCAAAATGCCAGAG<br>GGGTGGGTTGGAAATGAAC	110
m-Myc	NM_010849	F<br>R	AGAGCTCCTCGAGCTGTTTG<br>TGAAGTTCACGTTGAGGGG	91
mTRAP	NM_001102405	F<br>R	CTGGAGTGCACGATGCCAGCGACA<br>TCCGTGCTCGGCGATGGACCAGA	419
mFos	NM_010234	F	CCAGTCAAGAGCATCAGCAA	247

		R	AAGTAGTGCAGCCCGGAGTA	
mCathK	NM_007802	F<br>R	CTTCCAATACGTGCAGCAGA<br>TCTTCAGGGCTTTCTCGTTC	155
mNFATc1	NM_016791	F<br>R	CCGTTGCTTCCAGAAAATAACA<br>TGTGGGATGTGAACTCGGAA	152
mDANCR	NR_145131	F<br>R	CCTCTCCCGGATGGCTGTAT<br>AGCCATTCAGTCACGGGTTT	162
mGAPDH	NM_001289726	F<br>R	CGTCCCGTAGACAAAATGGT<br>TTGATGGCAACAATCTCCAC	110

“m” stands for “mouse”.
